# Localization of Microfibrillar-Associated Protein 4 (MFAP4) in Human Tissues: Clinical Evaluation of Serum MFAP4 and Its Association with Various Cardiovascular Conditions

**DOI:** 10.1371/journal.pone.0082243

**Published:** 2013-12-13

**Authors:** Helle Wulf-Johansson, Sofie Lock Johansson, Anders Schlosser, Anne Trommelholt Holm, Lars Melholt Rasmussen, Hans Mickley, Axel C. P. Diederichsen, Henrik Munkholm, Tina Svenstrup Poulsen, Ida Tornøe, Vicki Nielsen, Niels Marcussen, Jørgen Vestbo, Susanne Gjørup Sækmose, Uffe Holmskov, Grith Lykke Sorensen

**Affiliations:** 1 Cardiovascular and Renal Research, Institute of Molecular Medicine, University of Southern Denmark, Odense, Denmark; 2 Centre for Individualized Medicine in Arterial Diseases (CIMA), Department of Clinical Biochemistry and Pharmacology, Odense University Hospital, Odense, Denmark; 3 Odense Patient data Explorative Network (OPEN), Odense University Hospital, Odense, Denmark; 4 Department of Cardiology, Odense University Hospital, Odense, Denmark; 5 Department of Cardiology, Lillebælt Hospital Vejle, Vejle, Denmark; 6 Department of Clinical Pathology, Odense University Hospital, Odense, Denmark; 7 Department of Respiratory Medicine, Odense University Hospital, Odense, Denmark; 8 Respiratory and Allergy Research Group, Manchester Academic Health Sciences Centre, University Hospital South Manchester NHS Foundation Trust, Manchester, United Kingdom; 9 Department of Clinical Immunology, Næstved Hospital, Næstved, Denmark; King’s College London School of Medicine, United Kingdom

## Abstract

Microfibrillar-associated protein 4 (MFAP4) is located in the extracellular matrix (ECM). We sought to identify tissues with high levels of MFAP4 mRNA and MFAP4 protein expression. Moreover, we aimed to evaluate the significance of MFAP4 as a marker of cardiovascular disease (CVD) and to correlate MFAP4 with other known ECM markers, such as fibulin-1, osteoprotegerin (OPG), and osteopontin (OPN). Quantitative real-time PCR demonstrated that MFAP4 mRNA was more highly expressed in the heart, lung, and intestine than in other elastic tissues. Immunohistochemical studies demonstrated high levels of MFAP4 protein mainly at sites rich in elastic fibers and within blood vessels in all tissues investigated. The AlphaLISA technique was used to determine serum MFAP4 levels in a clinical cohort of 172 patients consisting of 5 matched groups with varying degrees of CVD: 1: patients with ST elevation myocardial infarction (STEMI), 2: patients with non-STEMI, 3: patients destined for vascular surgery because of various atherosclerotic diseases (stable atherosclerotic disease), 4: apparently healthy individuals with documented coronary artery calcification (CAC-positive), and 5: apparently healthy individuals without signs of coronary artery calcification (CAC-negative). Serum MFAP4 levels were significantly lower in patients with stable atherosclerotic disease than CAC-negative individuals (p<0.05). Furthermore, lower serum MFAP4 levels were present in patients with stable atherosclerotic disease compared with STEMI and non-STEMI patients (p<0.05). In patients with stable atherosclerotic disease, positive correlations between MFAP4 and both fibulin-1 (ρ = 0.50; p = 0.0244) and OPG (ρ = 0.62; p = 0.0014) were found. Together, these results indicate that MFAP4 is mainly located in elastic fibers and is highly expressed in blood vessels. The present study suggests that serum MFAP4 varies in groups of patients with different cardiovascular conditions. Further studies are warranted to describe the role of serum MFAP4 as a biomarker of stable atherosclerotic disease.

## Introduction

Microfibrillar-associated protein 4 (MFAP4) is a matricellular protein belonging to the fibrinogen-related protein superfamily. This family also includes fibroleukin, ficolins, FIBCD1, angiopoietins, and tenascins, which play multifaceted roles in innate immunity, the development of the cardiovascular system, and the normal functioning of the endothelium [1]–[5]. The MFAP4 gene consists of a signal peptide, a short N-terminal region comprising an Arg-Gly-Asp (RGD) sequence followed by the C-terminus [6]. The RGD sequence is the ligand motif for cell surface integrins and is associated with cell adhesive activity [7]. The MFAP4 protein is a disulfide-linked dimer that forms higher oligomeric structures [8].

MFAP4 has considerable sequence homology with the 36-kDa bovine microfibril-associated glycoprotein (MAGP-36), which was first discovered in porcine aorta and has since been detected in a wide variety of elastic tissues [9], [10]. Both MFAP4 and MAGP-36 bind to elastin and collagen fibers [8], [11]–[13]. Elastic fibers and collagen fibers are components of the extracellular matrix (ECM) that ensure the structural integrity of the ECM by maintaining the elasticity in the arterial wall, lungs, skin, and other dynamic connective tissues [14]. The biological function of MFAP4 is not fully documented; however, MFAP4 may interact with fibrillin-1 in dermal tissue, suggesting a role for MFAP4 in skin homeostasis during skin photobleaching [12]. MFAP4 has been proposed as a new candidate gene for left-sided congenital heart syndrome [15], and using proteome analysis, MFAP4 expression has been associated with aortic aneurysms [16], [17], pulmonary hypertension [18] and cirrhotic disease [19]. In cirrhotic tissues, MFAP4 synthesis is associated with ECM remodeling, and the accumulation of MFAP4 was observed in cirrhotic tissues and in the circulation of cirrhotic patients, suggesting measurements of serum MFAP4 levels as a biomarker for cirrhotic disease [19].

Altered vascular and cardiac ECM remodeling is evident in cardiovascular diseases (CVD) [20]. The use of biomarkers in CVD has become increasingly important because pathology-related vascular and cardiac remodeling is initiated before the appearance of clinical symptoms [21]. Biomarkers that reflect abnormal remodeling might be beneficial for the early diagnosis of CVD, and circulating proteins from ECM turnover are thought to identify such changes [22], [23].

In the current study, we initially aimed to establish the presence and localization of MFAP4 in human tissues. We used molecular biology techniques to characterize the gene expression profile of MFAP4 in various normal human tissues. qPCR was used to compare the mRNA expression levels and immunohistochemistry analysis was performed to investigate the localization of the MFAP4 protein. Cell culture experiments were implemented to determine relevant cell types synthesizing MFAP4. Subsequently, we evaluated whether the amount of serum MFAP4 is associated with different degrees of atherosclerotic CVD in a clinical cohort (n = 172) with comparable gender and age profiles.

## Materials and Methods

### Production of Monoclonal Anti-MFAP4 Antibodies

C57BL6/N MFAP4-null mice were immunized for the production of monoclonal antibodies against MFAP4. In brief, the mice were immunized five times with approximately 10 µg of recombinant V5-His tagged MFAP4, produced as previously described [8], with two weeks between each immunization. The mice were boosted three times with 10 µg of recombinant V5-His-tagged MFAP4 diluted in 200 µl of PBS by injection into the dorsal tail vein. Mice were terminated by cervical dislocation and B-cell hybridomas were produced by fusion between splenocytes and myeloma cells (ATCC, CRL-2016, Sp2/mIl-6) as described previously [24]. Antibodies were purified using a HiTrap Protein G column (GE Healthcare cat. no. 17-0404-01) on ÄKTA FPLC system (GE Healthcare). Mice were housed in Macrolon type II cages. Cages contained aspen woodchip bedding (Tapvei, Brogaarden, Gentofte, Denmark) and nesting material (EnviroDri, Brogarden, Gentofte, Denmark). All mice had access to chow, Altromin 1324 (Altromin International, Germany), and water ad libitum. The environment was controlled with respect to temperature (21–24°C), humidity and illumination (12 hour light/dark cycle with a 30 minute sunrise and dawn function. The use of the C57Bl6/N MFAP4-null mice and production of monoclonal antibodies were performed under a license obtained from The national Animal Experiments Inspectorate.

### Cell Culture

In general, cells were grown at 37°C in a 5% CO_2_ humidified incubator (Hera cell, Heraeus). The human cardiomyocytes from Innoprot were cultured in Cardiac Myocyte Medium (Innoprot) for two days. Fetal human aorta vascular smooth muscle cells (VSMCs) (Cell Application, Inc.) derived from the normal human tunica intima and tunica media of fetal aortas were allowed to differentiate in a VSMC differentiating medium for ten days (Cell application, Inc.). Once the cell cultures reached 90% confluence the cell culture supernatants were removed and centrifuged at 1000×g for 10 min. Then, 0.05% azide was added, and the cell culture supernatant was stored at 4°C until further analysis. The cells were washed with phosphate-buffered saline (PBS) and used for RNA and protein analysis.

### Quantitative Real-time PCR (qPCR)

Total RNA was obtained from whole organs from various human tissues (Clontech, Palo Alto, CA). cDNA synthesis was performed from 1 µg of purified RNA using an oligo(dT)18 primer and the Superscript III reverse transcriptase according to the manufacturer’s recommendations (Invitrogen).

Total RNA was obtained from human cardiomyocytes and differentiating VSMCs using the RNeasy Mini Kit (Qiagen, Ballerup, Denmark). RNA extraction was quantified on a NanoDrop spectrophotometer by measurement of the optical density at 260 nm, and cDNA synthesis was performed from 1 µg of purified RNA using the QuantiTect Reverse Transcription kit according to the manufacturer’s recommendations (Qiagen).

The Taqman primers and probes for MFAP4 (Hs00412974_m1) and β-actin (Hs99999903_m1) genes were obtained from Applied Biosystems. Analysis was performed in triplicate using cycles of 50°C for 2 min and 95°C for 10 min, followed by 40 cycles of 95°C for 15 s and 60°C for 1 min on a 7500 Real-Time PCR System (Applied Biosystems). The normalized quantity of MFAP4 was calculated as 2^−ΔCt^, where Δ*C*_t_ was obtained directly by subtracting *C*_t_ for MFAP4 from *C*_t_ for the β-actin gene.

### Ethics Statement

Human tissues were used for analysis by immunohistochemistry. Human healthy tissues and tissues from patients with acute myocardial infarction (AMI) were obtained from the diagnostic Biobank at the Department of Pathology, Odense University Hospital (Odense, Denmark). The Regional Scientific Ethical Committee for Southern Denmark approved the use of the healthy human tissue sections for research purposes (Ref. No VF20050070) and samples were obtained from patients with written informed consent. Moreover, the Regional Scientific Ethical Committee for Southern Denmark approved the use of diseased tissue sections with acute myocardial infarction (AMI) (S-20130056).

### Immunohistochemistry Procedure

Immunohistochemical staining was carried out on a Dako Autostainer Universal Staining System (Dako, Denmark A/S, Glostrup, Denmark). The 4-µm paraffin sections were dewaxed with xylene and rehydrated through a gradient of alcohols. Endogenous biotin reactivity was blocked with 1.5% hydrogen peroxide. Epitope retrieval was performed by protease treatment (0.05% protease type XIV; Sigma-Aldrich, St Louis, MO). Sections were then incubated for 60 min with monoclonal anti-MFAP4 (HG-HYB7-14) antibody diluted in TNT Antibody Diluent (Dako Denmark A/S). Visualization of the antigen–antibody complex was carried out with the PowerVision+ (Leica, Denmark A/S) detections system according to the manufacturer’s manual. DAB was used as a chromogen (K3468, Dako). Finally, sections were counterstained with Mayer’s hematoxylin (Bie & Berntsen, Herlev, Denmark) for 2 min, and coverslips were mounted with Aquatex (Merck, Darmstadt, Germany). The specificity of the immuno-staining was verified by omission of the primary antibody. Tissues were photographed with a Leica DMLB microscope and a Leica DFC 300 FX camera using the computer image analysis system LASV3.6.

### Protein Purification and Western Blotting

After removing the culture supernatant, VSMCs and cardiomyocytes were washed in PBS, followed by adding 600 µl/flask of cold RIPA buffer consisting of TBS, 1% NP-40 (Sigma-Aldrich), 1% sodium deoxycholate (Sigma-Aldrich), 0.1% SDS, and a mini complete protease inhibitor cocktail tablet (Roche). Subsequently, the flask was put on ice and allowed to incubate on a shaking table for 20 min. The cell suspension was transferred to an Eppendorf tube and centrifuged at 10,000×g for 10 min. The supernatant was transferred to a new Eppendorf tube and stored at −20°C until further protein analysis. SDS-PAGE samples were mixed with SDS-PAGE buffer (40% (v/v) glycerol, 8% (W,v) SDS, 25% (v/v) 4x lower Tris (pH 8.8), 0.002% brophenol blue) and added 0.02 M iodoacetamide (IAA). The samples were heated to 100°C for 1 min and finally alkylated by the of 1.4 M IAA. Protein lysates and culture supernatant were separated on 4–12% (w/v) polyacrylamide gradient gels (Criterion™ XT Precast Gel) with a discontinuous buffer system (MOPS SDS running buffer, NuPAGE, Invitrogen) and blotted onto a polyvinylidene difluoride membrane (PVDF) membrane (Amersham Hybond™-P). The pre-stained precision plus protein standards (Bio-Rad) marker was used.

Two membranes were prepared with either protein lysate or cell culture supernatant. The membranes were incubated with monoclonal anti-MFAP4 (HG-HYP 7-5) antibody diluted to 0.5 µg/ml in TBS, 0.1% (w/w) TWEEN 20 with 5% (w/v) non-fat milk (Bio-Rad) at 4°C overnight. In addition, the membrane with protein lysate was incubated with mouse monoclonal anti-GAPDH antibody (6C5, Santa Cruz Biotechnology) diluted to 1∶10,000. Next day, the membranes were washed and incubated with rabbit anti-mouse HRP-labeled immunoglobulin (Dako) diluted to 1∶20,000 in TBS containing 0.1% (w/w) TWEEN 20 and supplemented with 5% (w/v) nonfat dry milk for 1 h at RT. Membranes were washed and developed using the enhanced chemiluminescence ECL Plus™ kit following the manufacturer’s instructions (GE Healthcare) using the Optimax 2021 developer machine (Protec).

### Patients and Sample Collection

The assay was evaluated in four groups with different degrees of atherosclerotic heart disease, which were selected from larger studies, including the DEF-AMI, the Odense Artery Biobank, and the Danish Risk Score Study (DanRisk), that were actively enrolling patients during the same time period at Odense University Hospital in Denmark. The groups were chosen to investigate the associations between biomarkers and differences in the degree or type of ischemic heart disease. Each group was matched with regard to sex and age. Groups 1 and 2 included patients initially enrolled in the DEF-AMI [25], [26]. This study consecutively enrolled patients admitted with suspected myocardial infarction, and for the present study, 29 patients diagnosed with ST elevation myocardial infarction (STEMI) and 23 patients diagnosed with non-STEMI were selected. Blood sampling was performed within the first 24 h of symptom onset. Group 3 included patients from the Odense Artery Biobank, which collects spare arterial tissue and blood samples from coronary bypass and other operations. Of the included patients, 30 patients with stable atherosclerotic disease underwent elective coronary artery bypass grafting, carotid atherectomies, or other bypass surgery. The patients were enrolled prior to the operation. Blood samples were taken the day before the bypass operation. Groups 4 and 5 comprised individuals initially enrolled in the DanRisk study [27], a population-based survey of patients born in either 1949 or 1959 living in the southern region of Denmark. Persons with diagnosed CVD or diabetes mellitus were excluded from the study. All subjects in groups 4 and 5 underwent a cardiac CT scan with assessment of the coronary artery calcium score (Agatston score). Group 4 (CAC-positive) included persons with CAC (n = 30), defined as an Agatston score above the 90^th^ percentile for healthy individuals of the same age and gender (498–4220). Subjects in group 5 (CAC-negative) had no CAC (n = 60).

In all groups, blood samples were drawn in tubes with EDTA and centrifuged at 2,000×g for 10 min. Serum was stored at −80°C until biochemical analysis. The protocols were approved by the Regional Scientific Ethical Committee for Southern Denmark (S-20080140, S-20100044, and S-20090082) and were conducted in accordance with the Declaration of Helsinki. Written informed consent was obtained from each participant.

### Detection of Serum MFAP4 by the AlphaLISA Technique

Monoclonal anti-MFAP4 (HG-HYB 7–14) antibody was conjugated to AlphaLISA Acceptor beads (Perkin Elmer) at a concentration of 0.1 mg antibody/mg acceptor beads following the manufacturer’s instructions. Monoclonal anti-MFAP4 (HG-HYB 7–18) was modified by labeling with (+)-biotin N-hydroxysuccinimide ester (Sigma H1759) to permit binding to AlphaLISA streptavidin-coated donor beads. The AlphaLISA procedure was performed using 384-well microtiter plates (white opaque OptiPlate™ from Perkin Elmer) containing 5 µl of diluted serum (final dilution 1∶100), 2 nM biotinylated HG-HYB 7–18, and 10 µg/ml HG-HYB 7–14 conjugated to Acceptor beads in a total of 20 µl AlphaLISA®HiBlock Buffer (PerkinElmer). The reaction mixture was incubated at room temperature for 60 min. Streptavidin donor beads were then added to reach a final concentration of 40 µg/ml, and the plate was incubated at room temperature in the dark for another 30 min, after which time it was read on an EnVision reader (PerkinElmer) using the AlphaScreen protocol. Briefly, the AlphaScreen protocol used AlphaScreen label 384-well Packard OptiPlates and the AlphaScreen 570 emission filter, a flash/time ratio of 0.55, a measurement height of 1 mm, an excitation time of 0.18 s, and an emission time of 0.37 s.

The experiments were performed in duplicate except for the standards and quality controls, which were performed in quadruplicate. The duplicate sample covariance was accepted if it was ≤10%. Standards were prepared by the serial dilution of MFAP4 overexpressing CHO cell culture supernatant in AlphaLISA®HiBlock Buffer. The concentration of the standard was estimated by the spiking of a known concentration of rMFAP4 into the samples. Standards included serial dilutions from 8000 mU/ml to 7.8 mU/ml. The concentration of rMFAP4 was determined using Amino Acid Analysis by Alphalyse A/S (Odense, Denmark). When measured in serum, 1 U/ml = 38 ng/ml. Quality controls were prepared from human serum pools and from rMFAP4 pools. One pool was prepared to contain a low content of MFAP4 (QLow), and two pools were spiked with purified rMFAP4 (QMid) and (QHigh). The three quality controls were included in each plate. Interplate variation was accepted if all quality control measurements were within two standard deviations obtained from >10 consecutive runs.

### Quantification of Fibulin-1, Osteoprotegerin, and Osteopontin in Serum

The serum concentration of fibulin-1, OPG, and OPN was determined by modified sandwich immunoassays using Europium-labeled streptavidin and biotin-labeled secondary antibodies for detection. In the fibulin-1 assay, an affinity-purified polyclonal antibody was used for coating, and a monoclonal antibody was used for detection, together with placenta purified fibulin-1 as the standard, as previously described [28]. Ingredients for the modified sandwich immunoassays for OPG [29] and OPN were obtained from R&D systems (cat no. DY805 and DY1433).

### Statistical Analysis

Serum MFAP4 levels, stratified by groups, are presented as the median and 95% confidence intervals. Due to the non-normal distribution of serum MFAP4, approximation to normal distribution was achieved by logarithmic transformation (ln) before further statistical analysis. Comparison between groups was done using ANOVA with the Student t-test as the post hoc analysis. The association between continuous variables was evaluated by a pairwise correlation with Sidak-adjusted significance levels. The association between variables was evaluated with Spearman’s rank correlation. A p-value below 0.05 was considered statistically significant.

## Results

### MFAP4 mRNA Expression in Human Tissues

**Figure 1:**
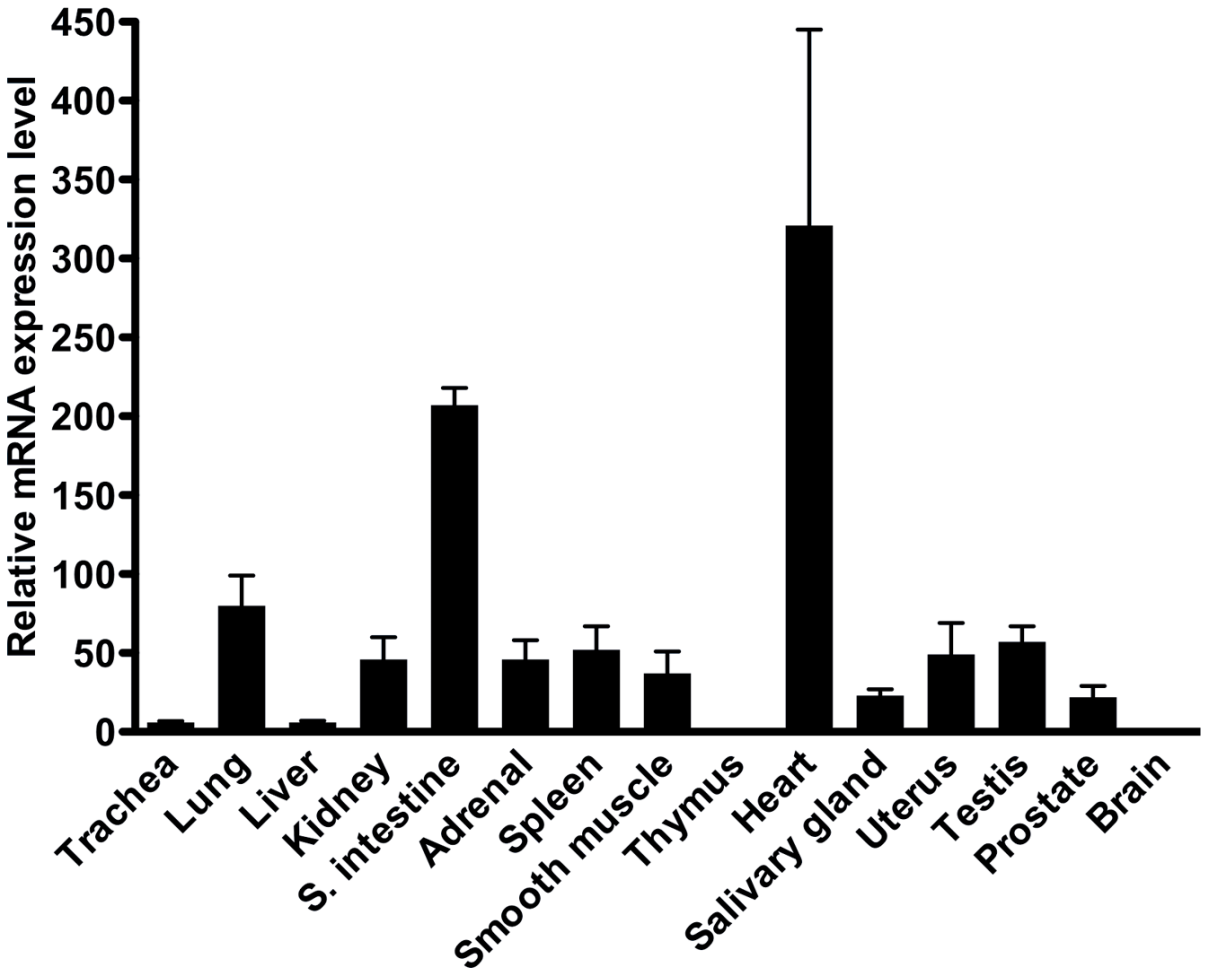
Quantitative real-time PCR analysis of MFAP4 mRNA transcripts in 15 different human tissues. High expression was detected in the heart, intestine, and lung. Data were normalized against β-actin. Results were calculated as mean+SD values from triplicate measurements. MFAP4 and β-actin mRNA transcripts were amplified by qPCR with gene-specific primers.

qPCR was performed in 15 different human tissues to determine MFAP4 mRNA transcript levels. Relative MFAP4 mRNA transcript levels were highest in the heart, small intestine, and lung, as shown in figure 1. MFAP4 expression was also observed in the kidney, testis, uterus, smooth muscle, spleen, and adrenal gland, whereas a low expression of MFAP4 mRNA transcripts was observed in the trachea, liver, salivary gland, and prostate. No detectable expression of MFAP4 was observed in the thymus and brain. All samples were normalized with respect to β-actin.

### Immunohistochemistry of MFAP4 Protein in Human Tissues

**Figure 2:**
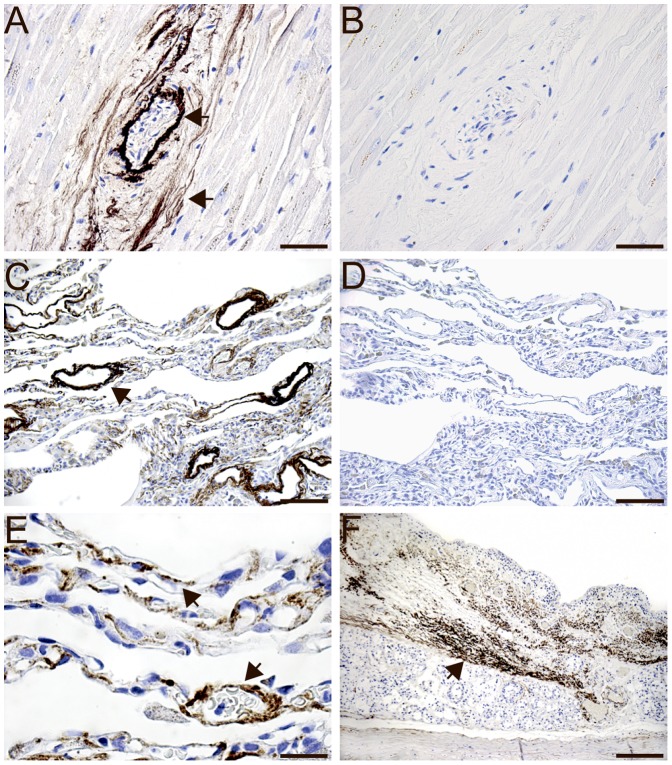
Immunohistochemical staining of MFAP4 in elastic fibers located in the blood vessels and the surrounding connecting blood vessels in the heart (A), in the pulmonary arterioles and interalveolar walls of the lung (C, E) as well as in the lamina propria of the trachea (F). Tissues were stained using the monoclonal anti-MFAP4 (HG-HYB 7–14) antibody and counterstained with Mayeŕs hematoxylin. Negative control sections with omission of the monoclonal anti-MFAP4 (HG-HYB 7–14) antibody in the heart (B) and in the lung (D) lung. *Scale bars:* (A, B) 50 µm, (C, D) 100 µm, (E) 20 µm and (F) 200 µm.

**Figure 3:**
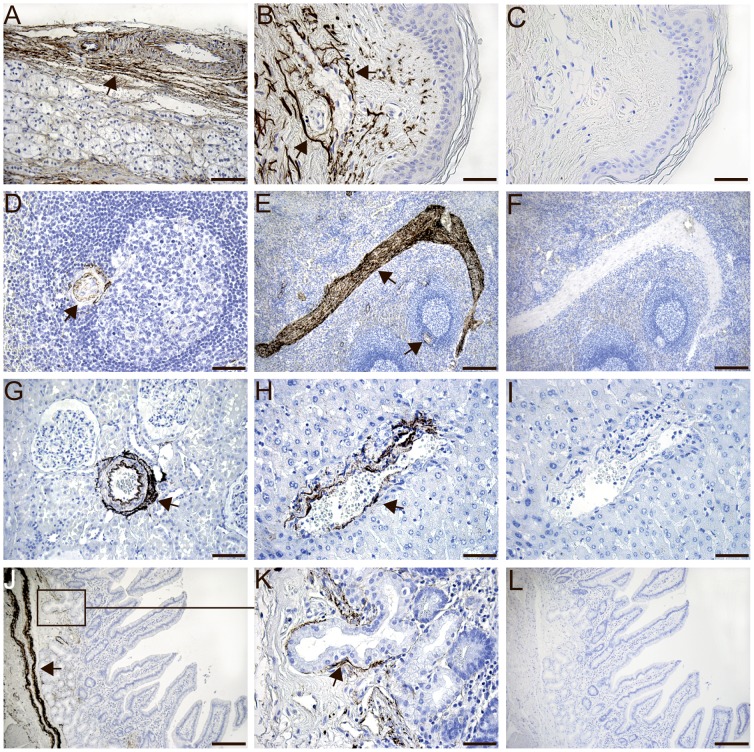
Immunohistochemical staining of MFAP4 located in elastic fibers in the adrenal gland (A), in the skin (B), in the central arteries and the trabeculae of the spleen (D, E), in blood vessels of the kidney (G), in the liver within blood vessels and the connective tissues in portal areas (H), in the blood vessels located in the submucosal layer and in the lamina propria and of the duodenum (J, K). Tissues were stained using the monoclonal anti-MFAP4 (HG-HYB 7–14) antibody and counterstained with Mayeŕs hematoxylin. Negative control sections with omission of the monoclonal anti-MFAP4 (HG-HYB 7–14) antibody in the skin (C), in the spleen (F), in the liver (I) and in the duodenum (L). *Scale bars:* (A, G) 100 µm, (B, C, E, H, I, K) 50 µm and (D, F, J, L) 200 µm.

**Figure 4:**
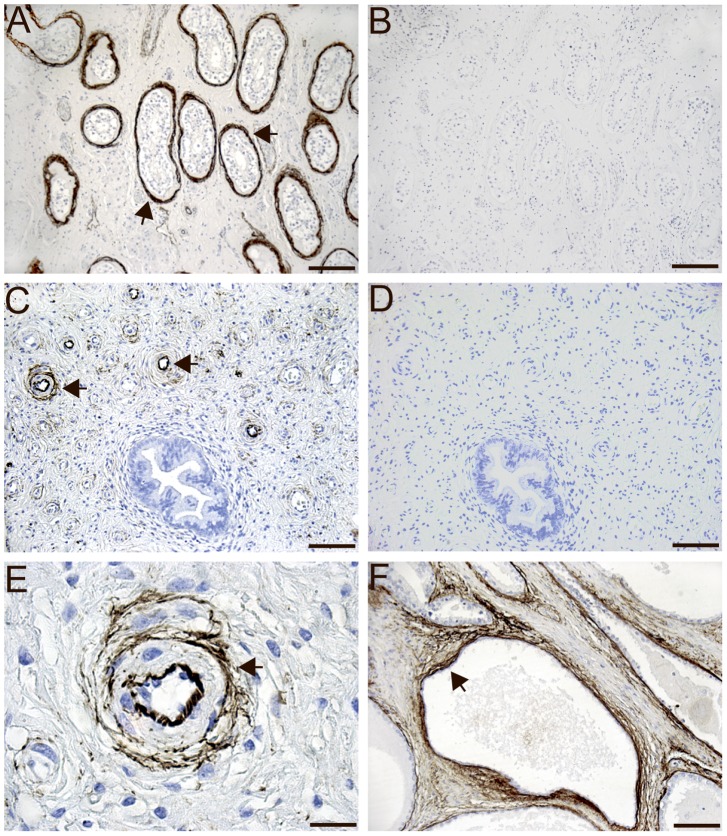
Immunohistochemical staining of MFAP4 in the basal membrane of the testis (A) and prostate (F) and in the spiral arteries of the uterus (C, E). Tissues were stained using the monoclonal anti-MFAP4 (HG-HYB 7–14) antibody and counterstained with Mayeŕs hematoxylin. Negative control sections with omission of the monoclonal anti-MFAP4 (HG-HYB 7–14) antibody in the prostate (B) and in the uterus (D). *Scale bars:* (A, B) 200 µm, (C, D, F) 100 µm and (E) 20 µm.

Human paraffin-embedded tissue sections were stained with the monoclonal MFAP4-antibody (HG-HYB 7–14) directed against human MFAP4. In the myocardium, MFAP4 was intensely detected in elastic fibers located within blood vessels and the surrounding connective tissue. No staining was seen in cardiomyocytes (Figure 2A). In the lungs, the intensive MFAP4 immunoreactivity was localized to the pulmonary arterioles (Figure 2C) and interalveolar walls (Figure 2E) but not in alveolar type I and type II cells. MFAP4 staining was also observed in the tela submucosa layer of the trachea (Figure 2F). MFAP4 staining of elastic fibers was observed in the adrenal gland (Figure 3A) and in the skin (Figure 3B). In the spleen, MFAP4 staining was localized to the central arteries (Figure 3D) and the trabeculae (Figure 3E). In the kidney, MFAP4 staining was localized to blood vessels (Figure 3G). In the liver, staining was seen in blood vessels and the connective tissues in portal areas (Figure 3H). In the duodenum, MFAP4 staining was intensely detected in blood vessels located in the submucosa layer (Figure 3J) and MFAP4 staining was observed in the lamina propria (Figure 3K). In the genitals, MFAP4 was visualized in the basal membrane of the testis (Figure 4A) and prostate (Figure 4F) as well as in the spiral arteries of the uterus (Figure 4, C and E). In negative control sections with omission of the primary antibody, no staining was observed. In all sections, MFAP4 staining appeared extracellularly and was associated with ECM fibrils.

### MFAP4 Synthesizing Cells

**Figure 5:**
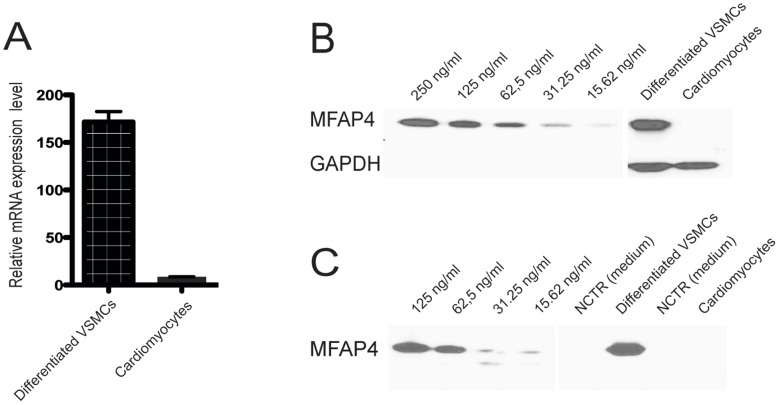
MFAP4-expressing cells. (A) qPCR analysis of MFAP4 mRNA transcripts in differentiating/contractile VSMCs and cardiomyocytes. MFAP4 mRNA transcripts were highly expressed by differentiating/contractile VSMCs. The data were normalized against β-actin. MFAP4 and β-actin mRNA transcripts were amplified by qPCR with gene-specific primers. (B) SDS-PAGE Western blot analysis using the monoclonal anti-MFAP4 (HG-HYP 7-5) antibody on protein lysate from differentiating/contractile VSMCs and cardiomyocytes. MFAP4 protein was strongly expressed in differentiated/contractile VSMCs as observed by its unreduced state of 66 kDa. Cell culture medium was used as a negative control (NCTR). GAPDH was used as a loading control. (C) SDS-PAGE Western blot analysis using the monoclonal anti-MFAP4 (HG-HYP 7-5) antibody on cell culture supernatant proteins from differentiating/contractile VSMCs and cardiomyocytes. MFAP4 was found in the culture supernatant from differentiated/contractile VSMCs.

We investigated whether MFAP4 protein synthesis was produced by VSMCs or/and cardiomyocytes. We included differentiated/contractile human VSMCs and human cardiomyocytes. qPCR revealed that MFAP4 mRNA transcripts were strongly expressed by differentiated/contractile VSMCs, whereas in cardiomyocytes, a low expression profile was observed (Figure 5A). Using protein lysate from the cells, a band of 66 kDa in the unreduced state was detected for MFAP4 in differentiated/contractile VSMCs using Western blot analysis (Figure 5B). Next, we analyzed the cell culture supernatant from the cell cultures and found that MFAP4 was detected in the cell culture supernatant from differentiated/contractile VSMCs (Figure 5C). The data indicate that MFAP4 expression is primarily synthesized from VSMCs and that cardiomyocytes exhibit a limited MFAP4 synthesis.

### Demographics

Demographic information and biochemical measurements are presented in Table 1.

**Table 1:** Demographics of the study population.

	STEMI^1^	Non-STEMI^2^	Stable atheroscleroticdisease^3^	CAC-positive^4^	CAC-negative^5^
	(n = 29)	(n = 23)	(n = 30)	(n = 30)	(n = 60)
Gender, male n (%)	23 (79%)	19 (83%)	23 (77%)	23 male (77%)	46 (77%)
Age, years	59.9±5.5	65.2±8.7	59.7±2.9	60.3±0.3	60.3±0.4
Total cholesterol (mmol/l)	4.7±1.7	4.8±1.3	4.5±1.3	5.5±1.2	5.4±1.0
Hypertension					
Male (%)	31	43	27	40	40
Female (%)	69	57	73	60	60
Smoking history					
Current (%)	45	26	57	43	25
Former (%)	34	39	30	40	55
Never (%)	21	35	13	17	20

^1^ Patients with ST elevation myocardial infarction (STEMI).

^2^ Patients with non-STEMI.

^3^ Patients destined for vascular surgery because of various atherosclerotic diseases (stable atherosclerotic disease).

^4^ Apparently healthy individuals with documented coronary artery calcification (CAC-positive).

^5^ Apparently healthy individuals without signs of coronary artery calcification (CAC-negative).

± standard deviation. Mean values

### Serum MFAP4 Levels in the Systemic Circulation after AMI Injury

**Figure 6:**
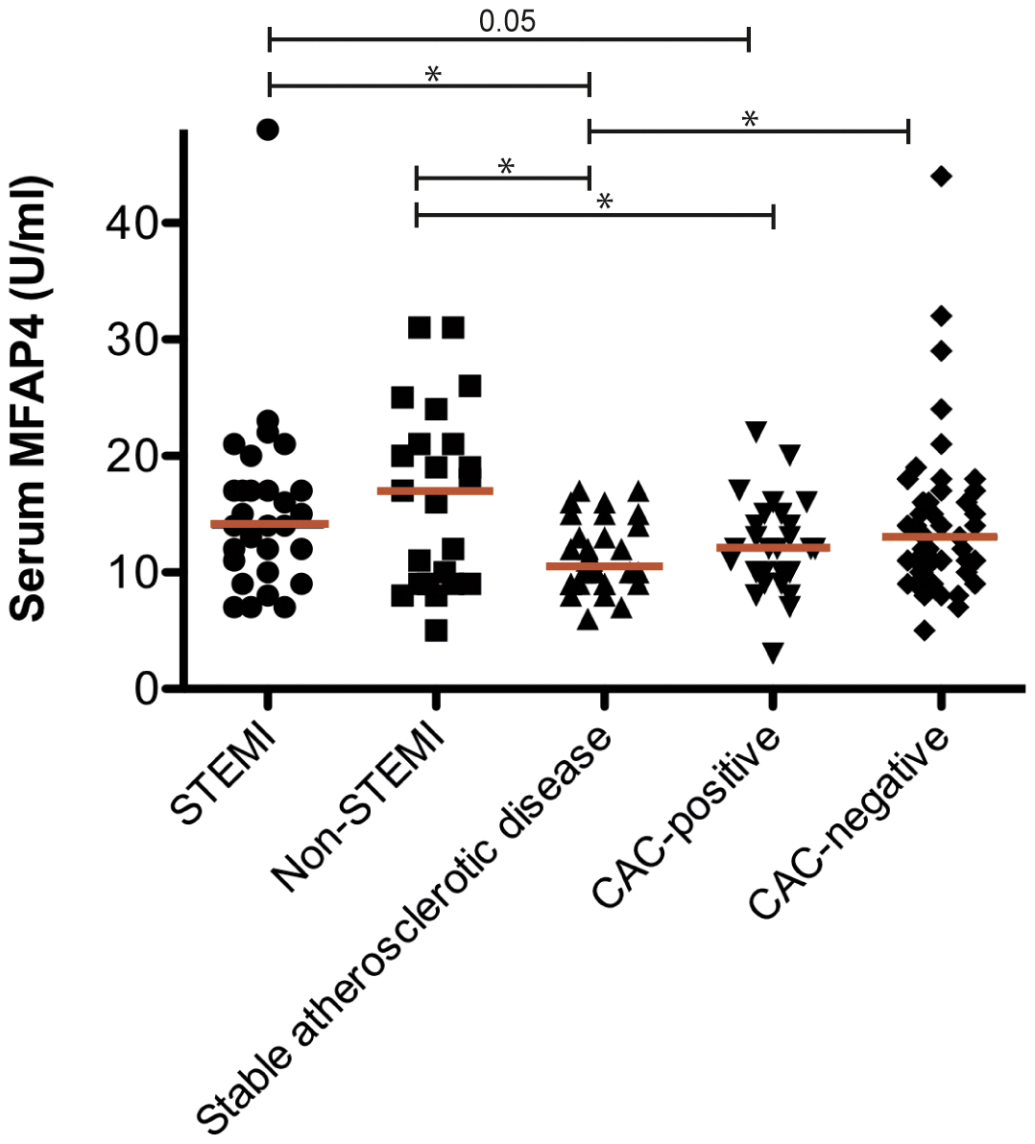
Systemic levels of MFAP4 measured in a clinical cohort with varying degrees of atherosclerotic CVD: 1: STEMI patients (n = 29); 2: non-STEMI patients (n = 23); 3: patients with stable atherosclerotic disease who underwent coronary artery bypass grafting, carotid atherectomies, or other bypass surgery for atherosclerotic diseases (n = 30); 4: apparently healthy individuals with documented coronary artery calcification (CAC-positive) (Agatston score>400) (n = 30); and 5: apparently healthy individuals without coronary artery calcification (CAC-negative) (Agatston score<400) (n = 60). A statistically significant difference between groups was measured by Student’s t-test (P<0.05). The bar shows the median value.

Recent work has revealed that MFAP4 is present in serum [19], and because we observed MFAP4 to be highly expressed in VSMCs, we sought to investigate whether serum MFAP4 could reflect ECM turnover in blood vessels in a clinical cohort with different degrees of atherosclerotic CVD. The serum MFAP4 median value with 95% confidence intervals was 14 [12]; [17] U/ml in the STEMI group, 17 [9]; [21] U/ml in the non-STEMI group, 10.5 [9]; [13] U/ml in the stable atherosclerotic disease group, 12 [10]; [13] U/ml in CAC-positive individuals, and 13 [12]; [14] U/ml in CAC-negative individuals (Figure 6). ANOVA demonstrated that MFAP4 varied significantly among the patient groups. Confounding variables such as gender, cigarette smoking status, and hypertension did not affect this relation. Serum MFAP4 levels were significantly lower in stable atherosclerotic disease patients compared with CAC-negative individuals (p<0.05). Furthermore, serum MFAP4 was lower in patients with stable atherosclerotic disease compared with patients with STEMI and non-STEMI (p<0.05). In addition, a significant difference was observed in non-STEMI compared with CAC-positive patients (p<0.05). A borderline significance was observed in STEMI patients compared with CAC-positive individuals (p = 0.05).

### MFAP4 in Myocardial Injury

**Figure 7:**
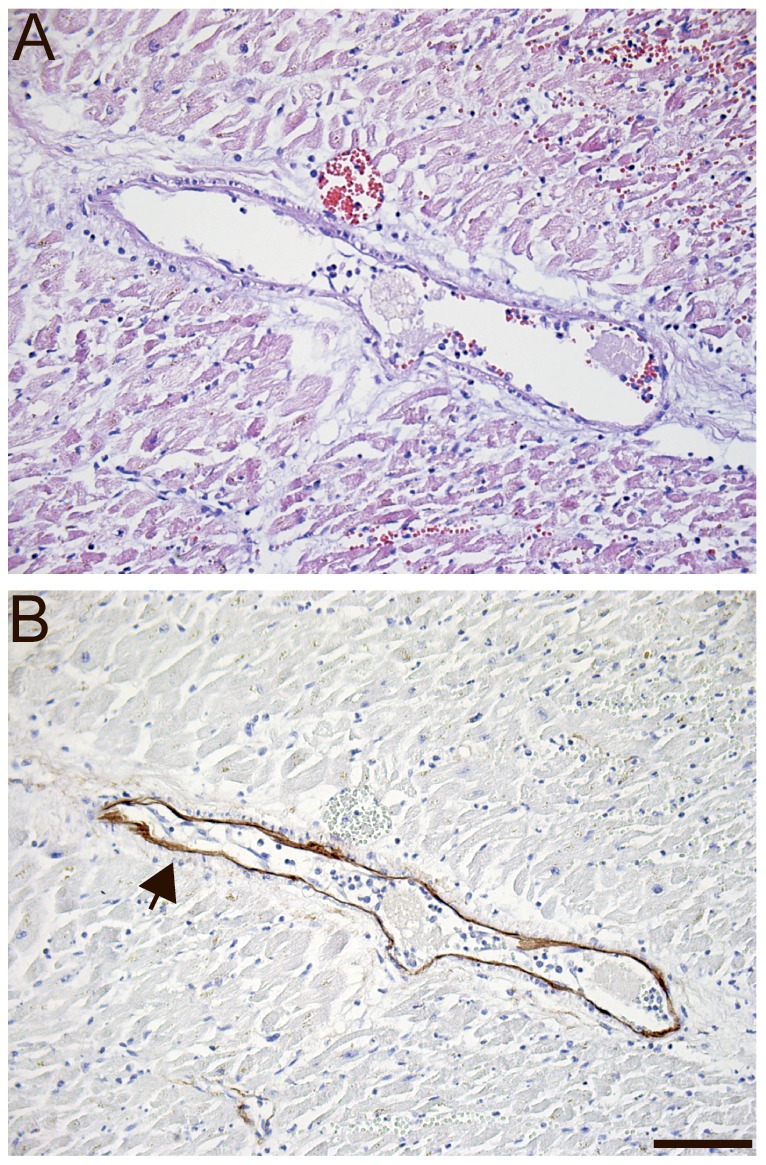
Histological section of a human heart from an area with myocardial infarction and counterstained with Mayeŕs hematoxylin (A). Immunohistochemical staining using the monoclonal anti-MFAP4 (HG-HYB7–14) antibody and counterstained with Mayeŕs hematoxylin (B). Staining of an infarcted area demonstrating MFAP4 staining within a blood vessel and no staining of cardiomyocytes. *Scale bars:* (A, B) 100 µm.

To investigate whether cardiomyocytes were able to synthesize MFAP4 during myocardial necrosis, paraffin-embedded sections from patients with AMI were included. Histological examination revealed that cardiomyocytes in the infarcted area showed nucleus disruption and provided evidence for white blood cell infiltration (Figure 7A). MFAP4 staining was visualized to elastic fibers located within the blood vessel and the surrounding connective tissue, and no staining was detected in the cardiomyocytes (Figure 7B).

### Serum MFAP4 Levels Correlate with Fibulin-1, OPG, and OPN

We sought to further delineate the role of MFAP4 by a comparison of serum MFAP4 variation with that of fibulin-1, OPG, and OPN, which are known to be associated with CVD [25], [28], [30]–[32]. As indicated in Table 2, we observed a positive correlation between serum MFAP4 and fibulin-1 (ρ = 0.57; p = 0.0324), OPG (ρ = 0.74; p = 0.0005), and OPN (ρ = 0.65; p = 0.0060) in non-STEMI patients. In patients with stable atherosclerotic disease, positive correlations between MFAP4 and fibulin-1 (ρ = 0.50; p = 0.0244) and OPG (ρ = 0.62; p = 0.0014) were found. No information on fibulin-1, OPG, and OPN was available for STEMI patients.

**Table 2:** Spearman correlation analysis of serum MFAP4 and ECM proteins such as fibulin-1, OPG, and OPN.

Variable	ρ	p
**Non-STEMI patients (n = 23)**		
Fibulin-1	0.57	0.0324
OPG	0.74	0.0005
OPN	0.65	0.0060
**Stable atherosclerotic patients (n = 29)**		
Fibulin-1	0.50	0.0244
OPG	0.62	0.0014
OPN	0.24	0.7102
**CAC-positive patients (n = 30)**		
Fibulin-1	0.24	0.7421
OPG	0.11	0.9936
OPN	0.04	1.0
**CAC-negative patients (n = 30)**		
Fibulin-1	0.26	0.6475
OPG	0.61	0.0021
OPN	0.23	0.7702

MFAP4: microfibrillar-associated protein 4; ECM: extracellular matrix; OPG: osteoprotegerin; OPN: osteopontin; Non-STEMI: Non-ST elevation myocardial infarction; CAC: coronary artery calcification.

## Discussion

Mature elastic fibers consist of elastin and additional ECM proteins that aid in assembly and link the fibers to the surface of surrounding cells [33]–[35]. MFAP4 is an ECM protein that is thought to play a role in elastin fiber formation and MFAP4 has been associated with ECM remodeling processes during vascular injury [12], [17], [36]. However, localization of MFAP4 in human tissues has been characterized in a limited number of published studies [8], [12], [37], [38], and given the paucity of publications on human MFAP4, we sought to characterize this in further detail. Furthermore, we included a cohort with different degrees of atherosclerotic CVD to investigate whether MFAP4 may be released and detected in the circulation during CVD events.

To investigate the MFAP4 gene expression profile, MFAP4 mRNA transcripts were investigated in 15 human tissues by qPCR. MFAP4 mRNA transcripts were highly expressed in the heart, lung, and intestine. These findings are in agreement with a previous study using Northern blotting [6]. Weak expression was found in the trachea, liver, salivary gland, and prostate. In the thymus and brain, no MFAP4 staining was observed, which is in agreement with another study [6]. However, mRNA expression may not directly correlate to protein expression as not all mRNA transcripts are translated into protein. Therefore, immunohistochemistry was included to localize the MFAP4 protein in human tissues. When using the monoclonal anti-MFAP4 (HG-HYB 7–14) antibody, MFAP4 protein was visualized at sites rich in elastic fibers, as observed in the heart, lung, skin, adrenal gland, spleen, kidney, liver, testis, prostate, and uterus. MFAP4 staining was further visualized within blood vessels in all tissues investigated. We have previously shown that human MFAP4 is localized to the alveolar wall and to elastic fibers in pulmonary arteries using another monoclonal anti-MFAP4 antibody showing the same localization pattern as the MFAP4 antibody used in the present study [8]. MFAP4 expression has further been localized to the skin [12], [39], which is in agreement with our findings. Moreover, molecular biology techniques such as electron microscopy and Western blotting have demonstrated that the bovine MAGP-36 protein was accumulated in rat tissues such as the aorta, skin, kidney, spleen, and lung [9], [11], [13]. In the liver, MFAP4 staining has previously been shown in the walls of blood vessels in portal areas, which is in accordance with our finding. Thus, in histological cirrhotic liver sections, MFAP4 showed intense staining patterns, suggesting that hepatic stellate cells induce MFAP4 synthesis during liver injury [19].

Since MFAP4 was located within blood vessels in almost every tissue investigated, we aimed to verify whether MFAP4 synthesis in the heart was produced by VSMCs or cardiomyocytes. To address this issue, we included *in vitro* studies to clarify whether human VSMCs and/or human cardiomyocytes were able to synthesize MFAP4. Both qPCR analysis and Western blotting revealed that MFAP4 synthesis was mainly derived from VSMCs.

The role of MFAP4 in cardiovascular diseases is still unknown, and therefore we studied serum MFAP4 in a clinical cohort to establish a link between serum MFAP4 levels and different stages of atherosclerotic CVD. Lower serum MFAP4 levels were observed in patients with stable atherosclerotic disease compared with CAC-negative individuals. Furthermore, the range of serum MFAP4 levels of CAC-positive patients was between the serum ranges of patients with stable atherosclerotic disease and CAC-negative patients. This finding suggests that MFAP4 synthesis from VSMCs within calcified blood vessels is differently regulated based on the severity of the atherosclerotic state. Vascular calcification of blood vessels occurs in the intima or/and the medial layer [40]. Calcification in coronary arteries occurs mainly within the intima, and it was recently demonstrated by proteome analysis that MFAP4 was downregulated in biopsies from atherosclerotic coronary intima compared with nonatherosclerotic intima [36]. Because we observed lower amounts of serum MFAP4 in patients with stable atherosclerotic disease relative to CAC-positive patients, we can speculate that the calcification in the more severely diseased patient group is located both in the intima and in the media and interferes with MFAP4 synthesis in both layers of the vessel wall whereas calcification in coronary arteries mainly is located within the intima layer suggesting that MFAP4 synthesis may still be released from VSMCs in the media layer into the circulation. Another explanation for the depressed serum MFAP4 levels may be that elastase activity increases proportionally to the extent of atherosclerotic changes, thereby decreasing the elastin content in atherosclerotic blood vessels [41], [42], which may affect MFAP4 synthesis negatively as a result of the inadequate binding properties of MFAP4 to elastin in the ECM.

We observed increased serum MFAP4 levels in STEMI and non-STEMI patients relative to patients with stable atherosclerotic disease. We assume that CAC is present in STEMI and non-STEMI patients; however, compared with CAC-negative patients, MFAP4 levels increase to the same or greater levels. However, because MFAP4 is a matricellular protein, we investigated whether MFAP4 was upregulated in cardiomyocytes during myocardial necrosis, as previously described for the matricellular proteins OPN and OPG [25], [30]–[32]. Paraffin-embedded sections from AMI patients revealed that MFAP4 did not appear to be upregulated in cardiomyocytes during myocardial necrosis. Therefore, we suggest that the increased MFAP4 levels observed is released from the blood vessel wall into the circulation during myocardial ischemia, leveling out the serum MFAP4 depression in atherosclerotic patients.

In the present study, serum MFAP4 levels correlate with fibulin-1 in non-STEMI patients and patients with stable atherosclerotic disease. Fibulin-1 is expressed in elastin-containing fibers and is found in the heart and in atherosclerotic lesions [28], [43], [44]. A stronger and positive association was further observed between MFAP4 and OPG in non-STEMI patients and in patients with stable atherosclerotic disease. OPG is involved in atherogenesis and vascular calcification and is expressed in cardiomyocytes after AMI [25], [30], [31]. Moreover, serum MFAP4 levels correlated strongly with OPN in non-STEMI patients. OPN is an ECM protein and plays a role in the healing process after AMI [32]. The data indicate that serum MFAP4 correlates with other cardiovascular risk markers, suggesting that MFAP4 may further expand the list of clinically assessed measures of ECM turnover. Fibulin, OPG, and OPN were not measured in the STEMI group.

It has previously been demonstrated that MFAP4 may serve as a biomarker for liver cirrhosis, where MFAP4 serum concentrations increase significantly with the progression of fibrosis development [19]. In fibrotic conditions, cell insensitivity to normal regulatory signals leads to excessive ECM deposition, and we can therefore only speculate that MFAP4 synthesis or ECM turnover is regulated differently in different organs or diseases, as MFAP4 levels were decreased in patients with stable atherosclerotic disease who were expected to have significant cardiovascular fibrosis.

The present findings must be interpreted in the context of several limitations. First, the sample size is small, and additional investigations with larger cohorts of healthy individuals and patients are needed to extensively evaluate MFAP4 as a potential biomarker in comparison with other CVD biomarkers. Secondly, the MFAP4 biomarker information was limited to a single measurement point, and monitoring heart injury might be achieved by using information from biomarker values over time. Third, the data indicate that serum MFAP4 levels could divide non-STEMI patients into two distinct groups. Non-STEMI patients represent a more heterogeneous group than STEMI patients, consisting of relatively more type 2 myocardial infarction patients, but the majority of the patients are of type 1 myocardial infarction like STEMI patients. This heterogeneity may possibly provide an explanation for the apparent distribution of serum MFAP4 into two distinct groups in the non-STEMI patients and larger studies with more patients within each category and with more detailed information about the type of myocardial infarction are warranted.

## Conclusion

In this study, we demonstrated that the MFAP4 protein is mainly localized to elastin-containing fibers within blood vessels. *In vitro* studies verified MFAP4 synthesis from VSMCs. Serum MFAP4 is decreased in the systemic circulation in stable atherosclerotic disease patients compared with CAC-negative individuals and STEMI and non-STEMI patients. Furthermore, serum MFAP4 can be correlated with known ECM proteins, such as fibulin-1, OPG, and OPN. MFAP4 is a component of the vascular ECM, and the data suggest that serum MFAP4 reflects the severity of the degree of atherosclerosis/calcification meaning that MFAP4 synthesis from VSMCs might be suppressed when both the intima and medial layer is calcified. Moreover, alternative studies of the biomarker MFAP4 should include atherosclerotic disease as a confounding effect. Although further investigation of the distribution and function of MFAP4 in atherosclerosis as well as its relation to the ECM are warranted, this may be addressed in future studies.
